# A wavelet based data coupling method for spatial damage detection in beam-type structures

**DOI:** 10.1371/journal.pone.0290265

**Published:** 2023-08-28

**Authors:** Jianwei Zhao, Zhuo Zhou, Deqing Guan, Jia Guo

**Affiliations:** 1 Department of Civil Engineering, Changsha University of Science & Technology, Changsha, Hunan, China; 2 Department of Civil Engineering, Hunan City University, Yiyang, Hunan, China; Semnan University, ISLAMIC REPUBLIC OF IRAN

## Abstract

Spatial damage identification is of great significance in mechanical, aerospace, and civil engineering. In this study, a data coupling method based on continuous wavelet transform (CWT) is proposed to identify the spatial damage location of beam-type structures. The singularity of the wavelet coefficient can be used to identify the signal singularity, and data coupling method calculates the spatial location of the damage. Numerical simulations and experimental analyses of different type of beams with transfixion damage are carried out to evaluate the accuracy of the method. The results show that the wavelet based data coupling method (W-DCM) can identify the minimum 4.9% damage severity of fixed beam and continuous beam, and can also identify the damage of non-free end of cantilever beam. However, the 9.7% damage severity of the free end of the cantilever beam cannot be identified. It is also found that the W-DCM can effectively circumvent the problem of wavelet coefficients edge effect. This method and wavelet singularity are used to provide a solution to the problem of structural edge damage identification.

## 1 Introduction

The damage identification theory based on structural vibration is that the damage will cause changes in the physical characteristics of the structure, including stiffness, damping and mass, and thus cause changes in the modal characteristics of the structure, including modal damping, natural frequency and modal shape. The performance of damage detection largely relies on the choice of damage sensitive features. Based on this theory, in recent years, many scholars have analyzed the changes of structural vibration characteristics to identify damage, and achieved good identification effects [1–6]. Some scholars [7] use displacement modes based on natural frequencies to effectively identify the damage of complex structures. Feng [8] extracted a first-order mode shape from the vehicle induced displacement response, which was utilized as a damage index to determine damage location and quantitatively monitor the damage progression of bridges.

Wavelet analysis based structural damage identification methods have developed rapidly in recent years. The wavelet coefficient diagram can directly locate the damage position. Montanari [9] obtained the first three free vibration modes of cantilever beam and simply sup-ported beam with edge cracking through numerical calculation, and used continuous wavelet transform (CWT) to identify the damage position. Yang and Nagarajaiah [10] combined independent component analysis with wavelet transform for out-put-only damage identification, and the damage information hidden in wavelet-domain signals was clearly revealed by a sharp spike. Katunin [11] applied sixth-order B-spline wavelet to detect the damage of composite plates. Numerical results and experimental results show that high-order B-spline wavelet can improve the sensitivity and accuracy of damage detection and localisation. Cao et al. [12, 13] applied wavelet transform to curvature mode shapes to mitigate the effects of noise. Then the local singularities of the signal are enhanced by Teager energy operator. Using this method, slight and multiple cracks in beams can be detected even under high noise conditions. Shahsavari et al. [14] proposed a statistical method to detect low-level multiple damage in the beam. The continuous wavelet transform is first applied to the first mode shape. Then principal component analysis (PCA) was used to extract the damage index from wavelet coefficients. Once damage is detected, likelihood ratio tests are further performed to determine possible locations. Guo et al. [15, 16] uses wavelet transform and improved particle swarm optimization algorithm to identify the damage location and damage degree of the beam, and identify the non-uniform dam-age of the beam. Zhu et al. [17] employed the 1D CWT to detect cracks in functionally graded beams and estimated the damage extent by calculating the Lipschitz regulation of wavelet coefficient.

The above studies used one-dimensional(1D) wavelet transform to identify structural damage, while most of the structural damage identification methods based on two-dimensional(2D) wavelet transform identify the damage location of plate structures [18–20]. Muyideen Abdulkareem [21] proposed an approach based on two-dimensional(2D) wavelet transform to solve the boundary distortion problem and demonstrated it by numerical and experimental examples of square steel plates. Wei Xu [22] formulates the wavelet 2D modal curvatures by ameliorating the 2D modal curvature using the real and complex wavelet transform, and the complex wavelet 2D modal curvature can characterize the length, extent, and non-uniformity of non-uniform cracks. Yam et al. [23] created a damage index using the difference between a pair of 2D modal curvature for intact and damaged plates. The capability of this index to identify damage was demonstrated on cracked aluminum plates. Li et al. [24] developed two damage indices using 2D modal curvature, namely a bending moment index and a residual strain mode shape index, utilizing them to identify damage in a free-boundary plate. The results showed that damage indices could identify the defective area with a reduced thickness accurately. Yang et al. [25] utilized the 2D Fourier transform instead of the second order differentiation to produce the 2D modal curvature, by which noise interference could be effectively suppressed. Recently, by ameliorating the 2D modal curvature with the wavelet transform and Teager energy operator, Xu et al. [20] proposed an integrated method and successfully utilized it to characterize the presence, location, and shape of an X-shaped notch in a plate with the aid of the non-contact laser measurement. Zhou et al. [26, 27] proposed a modified 2D Morlet wavelet function in the purpose of making 2D CWT behave like a quasi-isotropic transform, and this method is verified by experimental test of impact damage identification in composite laminate.

For a certain length of data, the edge effect will appear during the wavelet transform, which is mainly manifested as the singularity of the boundary wavelet coefficient. At present, there are some solutions to reduce edge effect, including ex-tending original signal [28], filling zero [12, 20], extrapolating signal extension [29], truncating boundary signal [2] and other methods to avoid distortion of edge damage identification. However, these methods try to change the original signal to avoid boundary distortion, which will increase the complexity of calculation and loss of information, resulting in errors in damage detection and unreliable damage identification results. When the damage occurs at the boundary position, these methods remove the peak value at the signal end to overcome the singularity problem and fail to identify the edge damage.

In the field of civil engineering, it is difficult to locate the spatial damage location of the structure based on the one-dimensional(1D) and two-dimensional(2D) wavelet transform. In this paper, we proposed a data coupling method based on the spatial slicing method of wavelet transform for the problem of structural spatial damage identification, which can achieve the effect of spatial dimensionality reduction but not data degradation, so as to identify the spatial damage location of the structure. The effectiveness of the proposed wavelet based data coupling method (W-DCM) is verified through numerical simulation and experimental study of spatial damage identification of different beams containing transfixion damage.

## 2 Wavelet based data coupling method (W-DCM)

### 2.1 Strain mode

Strain is the first derivative of displacement, so each displacement mode corresponds to the corresponding strain mode, and the strain mode reflects the inherent characteristics of the structure. To measure strain, the curvature mode can be used for indirect measurement. According to the material mechanics, the bending static relation of the beam can be obtained as [15]

$$\rho_{i}=\frac{1}{d_{i}}=\frac{M_{i}}{E_{i}I_{i}},$$ (1)

where *i* is the section position of measuring point *i*, *M*_*i*_ is the bending moment of section *i*, *E*_*i*_*I*_*i*_ is the flexural rigidity of section *i*, *d*_*i*_ is the radius of curvature at section *i*, and *ρ*_*i*_ is the curvature of section *i*. According to the approximate equation of bending deformation of the beam [15],

$$\rho =\frac{d^{2}y}{dx^{2}},$$ (2)

where *x* is the coordinate along the length direction of the straight beam and *y* is the bending deflection of the beam. According to Eqs (1) and (2), the difference equation of three equidistant continuous measuring points along the beam can be obtained [16]:

$$\rho_{i}=\frac{M_{i}}{E_{i}I_{i}}=\frac{y_{i+1}-2y_{i}+y_{i-1}}{\Delta^{2}},$$ (3)

where *i*+1, *i*, and *i*−1 are three adjacent continuous mea-suring points with equal distance along the beam, *ρ*_*i*_ is the curvature of section *i*, *y*_*i*_ is the bending deflection of section *i*, *y*_*i*+1_ and *y*_*i*−1_ are the bending deflections of section *i*−1 and section *i*+1 of the measuring points, respectively, and Δ is the distance of two adjacent measuring points. The strain *ε*_*i*_ of the measuring point *i* of the beam can be expressed as [16]

$$\varepsilon_{i}=-\frac{h_{0}}{d_{i}}=-h_{0}\frac{y_{i+1}-2y_{i}+y_{i-1}}{\Delta^{2}}=-h_{0}\rho_{i},$$ (4)

where *h*_0_ is the distance between the surface of the measuring point on the beam and the neutral layer, and Eq (4) shows the direct relationship between the curvature mode and the strain mode of the beam.

### 2.2 Continuous wavelet transform based data coupling method (W-DCM)

In signal processing, three-dimensional(3D) wavelet transform cannot be displayed because the wavelet coefficients are six-dimensional, so the common solution is the spatial slicing method, which fixes one-dimension (1D) of the 3D data to carry out wavelet transform on other dimensions, so as to achieve the purpose of dimensionality reduction. In wavelet data coupling method(W-DCM), the dimensional fixation of 3D data is done by coupling the data in the direction of the fixed dimension to other dimensions, so as to obtain the purpose of " spatial dimensionality reduction but not data reduction [30]", which is more convenient for the identification of spatial damage of the structure.

In structural damage identification based on vibration signals, measuring points all contain multi-dimensional information. Therefore, in order to detect the 3D information of the structure, we need to obtain the 3D data of the structure first, and then solve the singularity problem of the 3D wavelet signal according to the singularity principle of wavelets.

**Fig 1:**
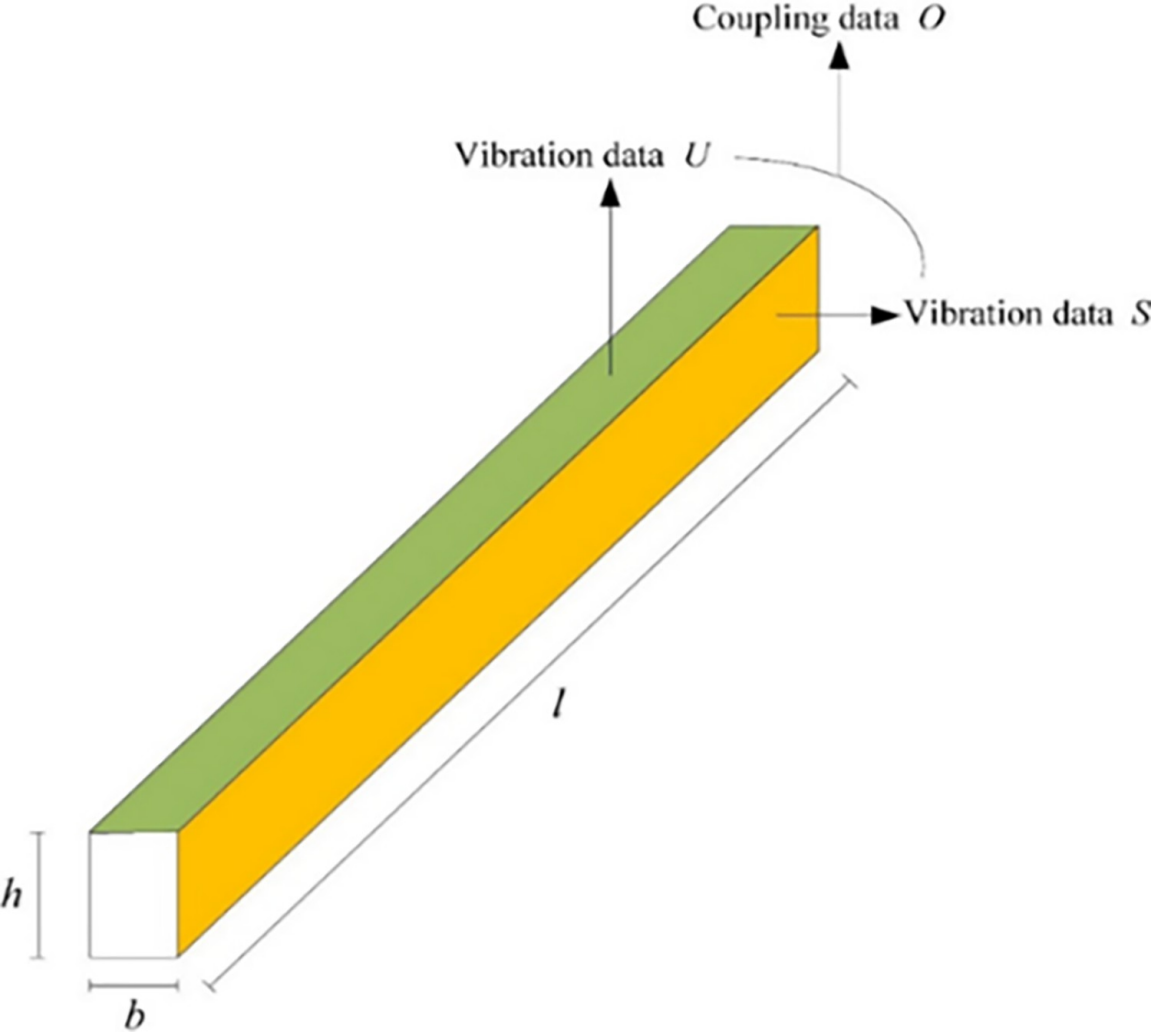
Coupling process of W-DCM.

**Fig 2:**
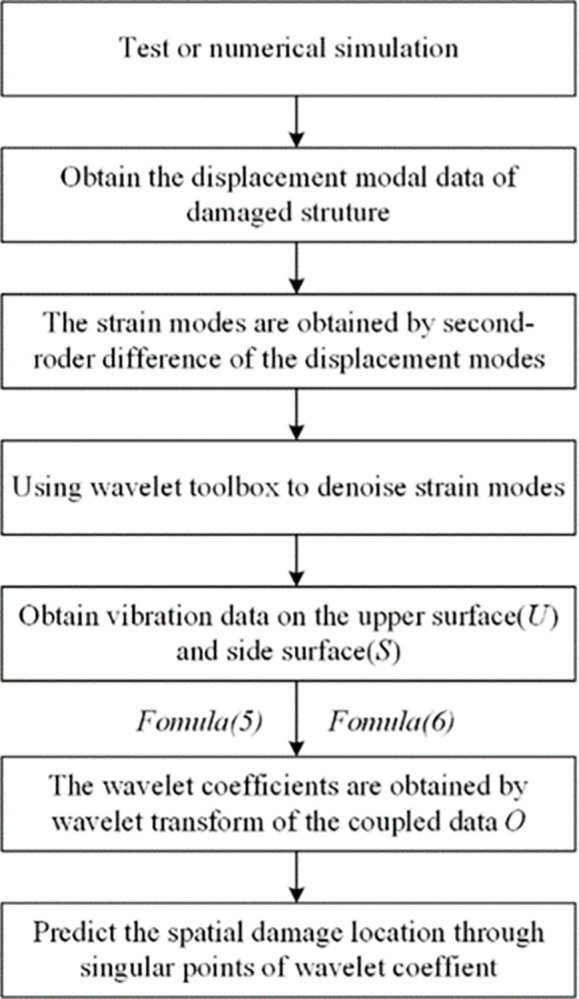
Flowchart of W-DCM based damage identification.

Using the above principle of data coupling method, taking the beam structure as an example, we can measure the vibration data *U* of the upper surface, and divide the side surface into *n* layers, then measure the vibration data *S* of each layer, and finally couple *U* with *S* to get the coupling data *O*, that is, to generate 3D vibration data. Due to the characteristics of the vibration data of the beam, the data in the length direction of the beam is much larger than the length of the data in the width direction (*l*≫*b*), and the data along the width direction *b* of the beam is too short to perform wavelet transform in numerical simulation and experiment, so the data in the width direction of the beam can be ignored and only the data in the length direction *l* of the upper surface of the beam is used. The process and flowchart of W-DCM is shown in Figs 1 and 2.

Taking the beam structure as an example, the vibration data of the upper surface of the beam structure in the length direction are first measured as *U*, and the data of the *i* layer of the beam side is *S*_*i*_, and the dimensions of *U* and *S*_*i*_ are the same. The first step is to normalize *S*_*i*_ into a dimensionless quantity *C*_*i*_ to facilitate the coupling of *U* with it. The normalization formula of *C*_*i*_ is

$$C_{i}(t)=\frac{S_{i}(t)}{S_{i}(max)}$$ (5)

In Eq (5), *C*_*i*_(*t*) denotes the *t* data in the *i* layer after normalization, *S*_*i*_(*max*) denotes the maximum value of the data in the *i* layer, and *S*_*i*_(*t*) denotes the *t* data in the *i* layer.

After obtaining the normalized matrix, coupling *U* with *C*_*i*_ to obtain the coupled data *O*_*i*_, whose coupling equation is

$$O_{i}(t)=U(t)\times C_{i}(t)$$ (6)

Where, *U*(*t*) denotes the *t* data in *U*, *O*_*i*_(*t*) denotes the *t* data of the *i* layer of coupling data. After getting the coupling data *O*_*i*_(*t*), wavelet transform is performed on *O*_*i*_(*t*) to get the wavelet coefficients, and then singularity is used to determine the damage location of the beam structure.

The 1D wavelet method employs *U* to identify the location of damage along the *l* direction, but does not identify where in the *h* direction the damage occurred. In the W-DCM, *C*_*i*_(*t*) amplifies the values at the damage location for each layer. Along the *h* direction, the closer to the damage location, the more pronounced the amplification effect of the values. The coupled values *O*_*i*_(*t*) are also subject to this amplification effect. After performing the wavelet transform on *O*_*i*_(*t*), the wavelet coefficients of *O*_1_~*O*_4_ are compared, and the layer with the largest wavelet singular value is the layer where the damage is located. The coupled values determine both the damage location along the *l* direction and the *h* direction, thus obtaining the spatial location of the damage.

## 3 Experimental analysis

**Fig 3:**
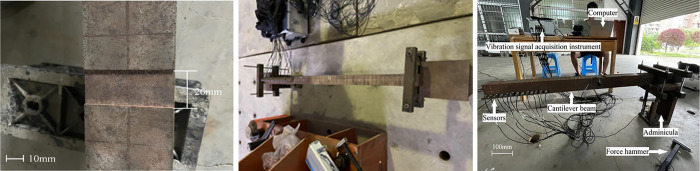
Beam test:(**a**) transfixion damage; (**b**) fixed beam; (**c**) cantilever beam.

The effectiveness and correctness of the above methods are proved by experimental analysis below. The research objects are fixed beam and cantilever beam as shown in section 3.1 and section 3.2 respectively. The damage is a notch with a width (26mm as shown in Fig 3A) and a depth (determined by damage severity). Fig 3 shows the experiment to obtain vibration data of fixed beam and cantilever beam. Table 1 shows the main parameters of beams.

**Table 1:** Parameters of beams.

Parameters	Value
Density(ρ)	7800kg/m^3^
Poisson’s ratio(μ)	0.3
Elasticity modulus(E)	2.1GPa
Material	Q235

There are 16 highly sensitive vibration sensors with a diameter of 22.5mm are used in the test to collect the displacement modal data of the beam. According to the designed scenarios, the beam is evenly divided into different number of elements along the length direction, with each element being 26mm in length. The side surface was equally divided into 4 layers, with each layer being 20mm in length. As the test was limited by the size of the vibration sensor, distance of 13mm was left at both ends as the installation position of the sensor. According to the different damage severity, the penetration damage of different depth is converted. Sensors were arranged on the element grid nodes, the test measured the overall first-order displacement modal data of the beam, and the damage severity was defined by stiffness reduction, namely:

$${EI}_{S}=EI\times (1-\gamma )$$ (7)

*EI* is the sectional stiffness of non-destructive beam, and *EI*_*S*_ is the stiffness of damaged section, γ is the damage severity. According to the stiffness formula and the parallel axis theorem of the beam with rectangular section, it can be obtained:

$${\left(h-d\right)}^{3}+3d^{2}(h-d)=h^{3}(1-\gamma )$$ (8)

*d* is the damage depth corresponding to the damage severity and *h* is the height of the beam section. Neglecting the complex roots of Eq (8), it can be obtained:

$$d=\frac{h}{2}({1-(1-2\gamma )}^{\frac{1}{3}})$$ (9)

According to Eq (9), the element damage depth corresponding to the severity of damage can be calculated.

### 3.1 Damage identification based on W-DCM of fixed beam

**Fig 4:**
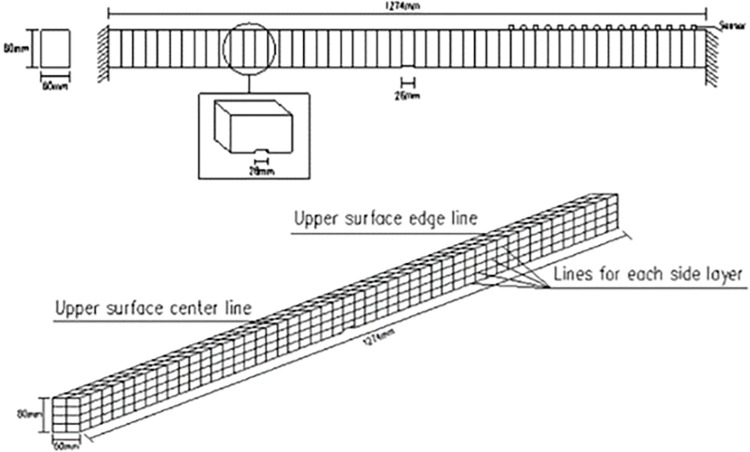
Meshing diagram of fixed beam: (**a**) sensor layout; (**b**) grid division.

Relevant parameters of fixed beam are as follows: span length of *l* = 1274*mm*, section size of *b*×*h* = 60*mm*×80*mm*, other parameters are shown in Table 1. There is one damage in scenario 1 and three damage in scenario 2 as shown in Table 2. The damage depth is calculated by Formula (9). The meshing diagram of fixed beam is shown in Fig 4.

**Table 2:** Scenarios of fixed beam with transfixion damage.

Scenarios	Damage element	Damage severity (γ) (%)	Damage depth *d* (mm)
1	25	18.6	5.73
2	12	4.9	1.35
	25	18.6	5.73
	49	9.7	2.76

**Fig 5:**
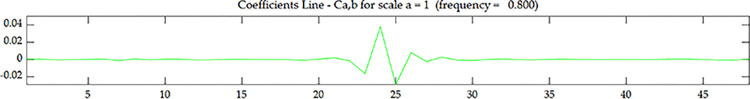
Wavelet coefficient of *U*^*C*^ under scenario 1.

**Fig 6:**
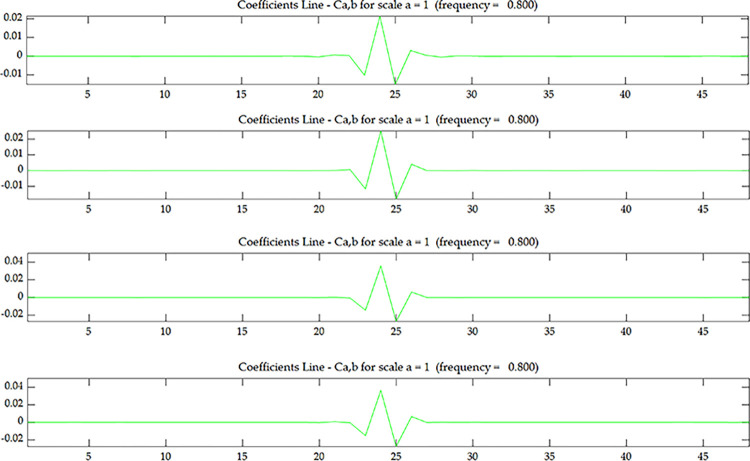
Wavelet coefficient of *O*_*1*_^*C*^~*O*_*4*_^*C*^ under scenario 1:(**a**) *O*_*1*_^*C*^; (**b**) *O*_*2*_^*C*^; (**c**) *O*_*3*_^*C*^; (**d**) *O*_*4*_^*C*^.

**Fig 7:**
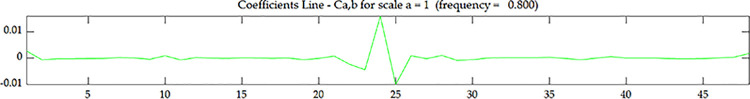
Wavelet coefficient of *U*^*E*^ under scenario 1.

**Fig 8:**
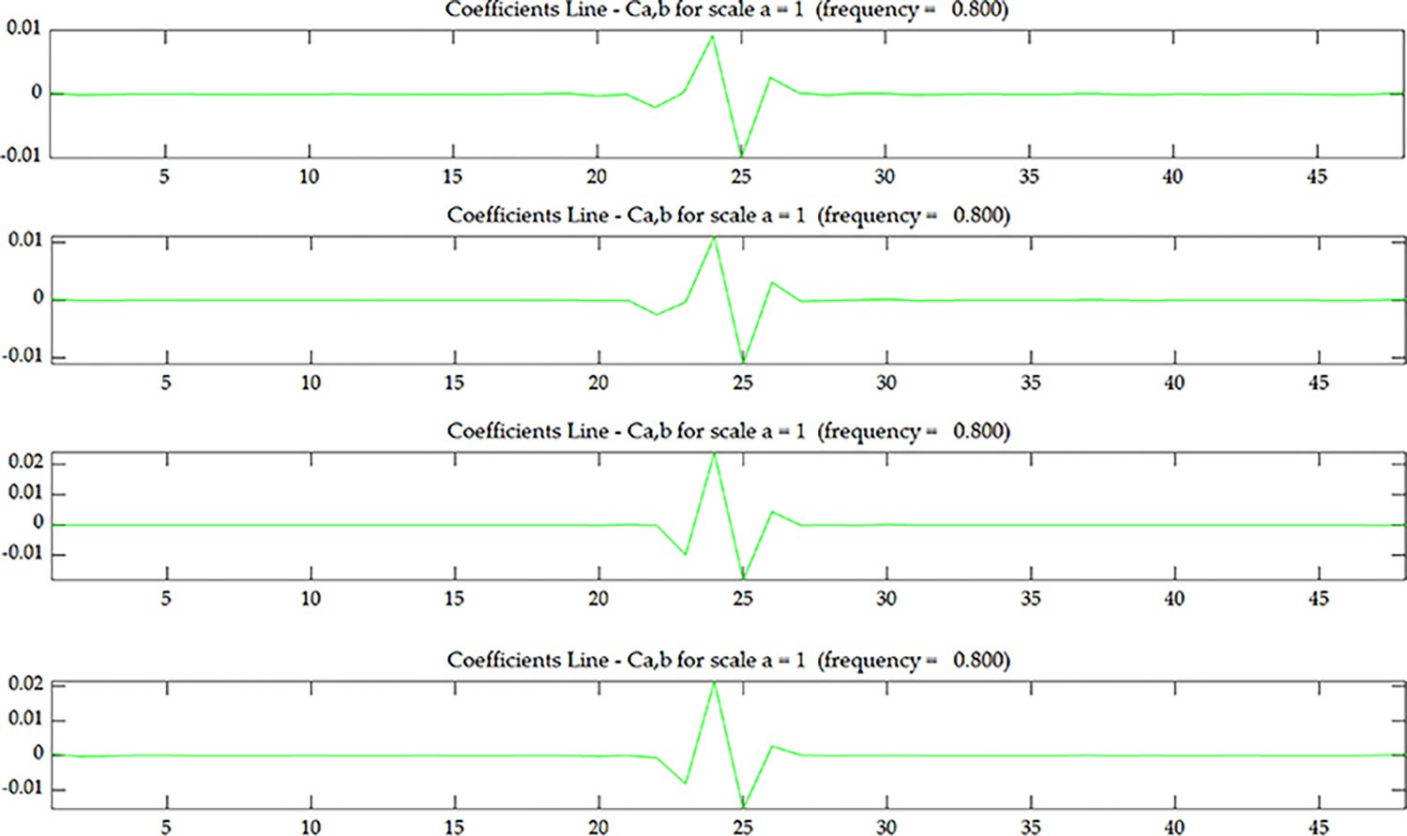
Wavelet coefficient of *O*_*1*_^*E*^~*O*_*4*_^*E*^ under scenario 1:(**a**) *O*_*1*_^*E*^; (**b**) *O*_*2*_^*E*^; (**c**) *O*_*3*_^*E*^; (**d**) *O*_*4*_^*E*^.

The wavelet coefficient of *U*^*C*^ is shown in Fig 5, and *U*^*C*^ is the strain modal data of the centerline of the upper surface. The wavelet coefficient of *U*^*E*^ is shown in Fig 7, and *U*^*E*^ is the strain modal data of the edge line of the upper surface. The wavelet coefficient of $O_{i}^{C}$ is shown in Fig 6, and $O_{i}^{C}$ is the data after coupling *U*^*C*^ with the strain mode of the *i* layer on the side (*S*_*i*_) according to Eqs (5) and (6). The wavelet coefficient of $O_{i}^{E}$ is shown in Fig 8, and $O_{i}^{E}$ is the data after coupling *U*^*E*^ with the strain mode of the *i* layer on the side (*S*_*i*_) according to Eqs (5) and (6).

From Figs 5 and 7, it can be seen that the wavelet coefficients in the middle of the beam span shows singularities, and the wavelet coefficients of the elements around the middle of the beam span are also affected to some extent. Based on the energy method [31], in addition to the stiffness of the damaged element itself will change, it will also cause the stiffness of several surrounding elements to change. Therefore, in addition to the singularity that occurs in the damaged element, there will be some fluctuations in the surrounding elements as well. From this, we can determine that the damage occurred in the middle of the beam span, but couldn’t determine where the damage occurred along the beam height. From Figs 6 and 8, as the coupling data gets closer to the damage layer, the wavelet coefficients of the middle of the beam get bigger. From this we can determine that the damage occurred at the bottom of the middle span. This shows that the data coupling method can identify single spatial damage location of fixed beam.

Comparing Figs 6 and 8, since the damage in this test is transfixion uniform damage, both the wavelet coefficient of $O_{i}^{C}$ and $O_{i}^{E}$ can identify the damage location, the subsequent test and numerical simulation only discuss wavelet coefficient of $O_{i}^{C}$.

**Fig 9:**
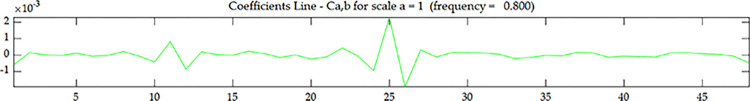
Wavelet coefficient of *U*^*C*^ under scenario 2.

**Fig 10:**
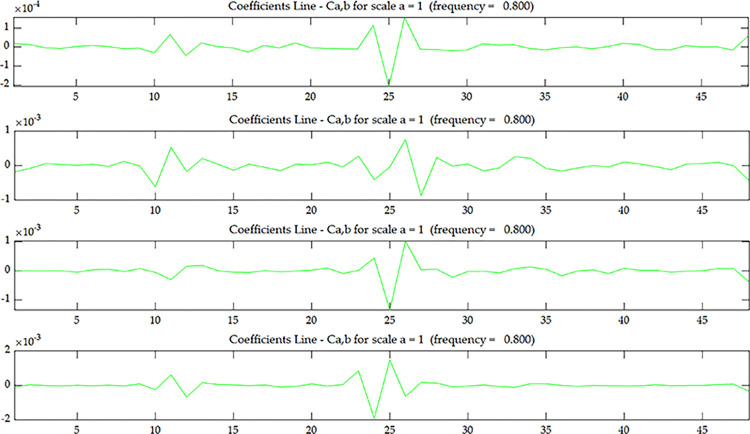
Wavelet coefficient of *O*_*1*_^*C*^~*O*_*4*_^*C*^ under scenario 2:(**a**) *O*_*1*_^*C*^; (**b**) *O*_*2*_^*C*^; (**c**) *O*_*3*_^*C*^; (**d**) *O*_*4*_^*C*^.

As can be seen from Fig 9, the wavelet coefficients of multi-damaged beams also have edge effect, which leads to the failure to judge whether there is damage at the edge. There are also singularities at element 12 and element 25, indicating the existence of damage in these two elements. After coupling the upper surface data with the side data, it can be seen from Fig 10 that the edge effect at the left end disappears, but the singularity still exists at the right end, indicating that the singularity at the left end of Fig 9 is caused by the edge effect, while the singularity at the right element 49 is caused by damage. Moreover, with the increase of coupling layers, the value of the wavelet coefficient at element 49 also increases, which indicates that the damage is located at the bottom layer of element 49.

It can also be found from Fig 10 that the coupled wavelet coefficient has singularities in both element 12 and element 25, and the value of the wavelet coefficient of the singularities increases with the number of coupling layers, indicating that the damage occurs at the bottom layer of element 12 and element 25. The singularity of the wavelet coefficients of the elements around the damage location is caused by the influence of the damage element on the stiffness of the elements around the damage location, rather than the damage of the elements around the damage location.

It can be proved through experiments that the W-DCM can effectively identify the spatial damage location of the fixed beam by using wavelet coefficient singularity, which on the basis of spatial dimensionality reduction but not data reduction, and the value of wavelet coefficients becomes larger as it gets closer to the damage location. These outcomes are as yet allowable when contrasted with the outcomes got in different works because the method not only obtains the spatial location of the damage [15, 16], but also does not require data from the undamaged state of the beam [32, 33].

### 3.2 Damage identification based on W-DCM of cantilever beam

**Fig 11:**
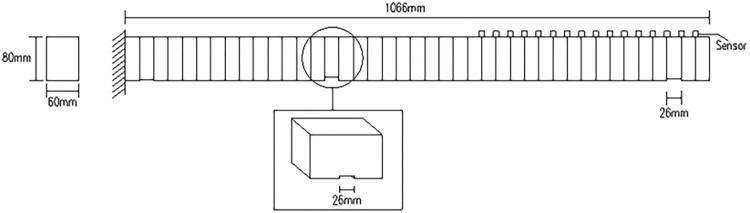
Meshing diagram of cantilever beam.

Relevant parameters of cantilever beam are as follows: span length of *l* = 1066*mm*, section size of *b*×*h* = 60*mm*×80*mm*,other parameters are shown in Table 1. The meshing diagram of cantilever beam is shown in Fig 11. The scenario 1 as shown in Table 3 and the damage depth is calculated by Formula (9).

**Table 3:** Scenarios of cantilever beam with transfixion damage.

Scenarios	Damage element	Damage severity (γ) (%)	Damage depth *d* (mm)
1	2	4.9	1.35
	15	18.6	5.73
	39	9.7	2.76

**Fig 12:**
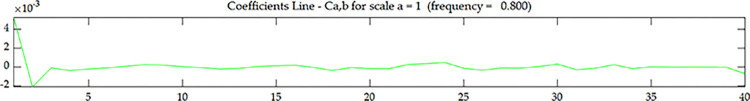
Wavelet coefficient of *U*^*C*^ under scenario 1.

As can be seen from Fig 12, the wavelet coefficient *U*^*C*^ of the cantilever beam with multiple damage also has edge effect, and it is impossible to judge whether there is damage at both ends of the beam. In addition, except for the existence of wavelet singularity at both ends due to edge effect, no singularity was observed in the rest parts, and the specific location of damage in the span could not be judged.

**Fig 13:**
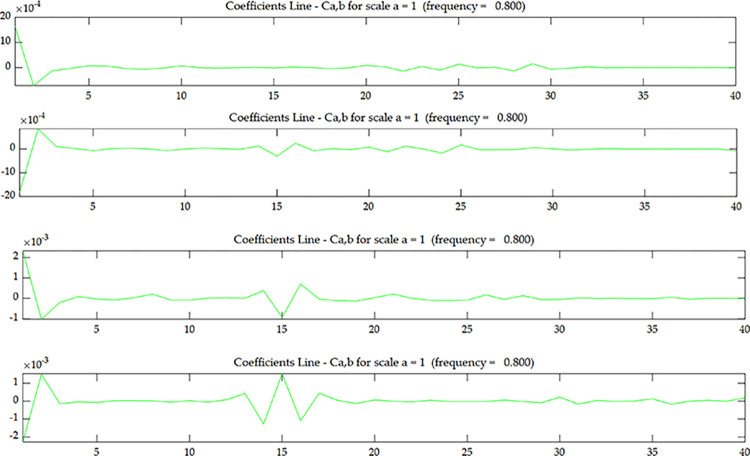
Wavelet coefficient of *O*_*1*_^*C*^~*O*_*4*_^*C*^ under scenario 1:(**a**) *O*_*1*_^*C*^; (**b**) *O*_*2*_^*C*^; (**c**) *O*_*3*_^*C*^; (**d**) *O*_*4*_^*C*^.

When the data is coupled, significant changes are observed. According to the previous test discussion of the fixed beam, the edge effect of the fixed end can be significantly reduced after data coupling. However, it can be seen from Fig 13 that there are always singular points at the fixed end, so it can be judged that there is damage at the fixed end. According to the test discussion of the fixed beam, if there is damage in element 1, then the coupled data will only generate one singular point at the end. However, as can be seen from Fig 13, there are 2~3 singular points in the coupled data at the fixedly supported end, which indicates that the damage location is not element 1, but element 2. As the coupling data gets closer and closer to the 4th layer, the wavelet coefficients of element 2 and element 15 also get larger and larger. Therefore, it can be judged that there is damage in element 2 and element 15, and the damage occurs at the bottom of the element.

According to the experimental design, there is a 2.76mm transfixion damage at the bottom of element 39 at the free end, but no obvious singularity is found in the wavelet coefficients of the upper surface data and the coupled data, which indicates that this method cannot effectively identify the damage at element 39 at the free end of the cantilever beam.

It can be proved through experiments that the W-DCM can identify the spatial damage location of the non-free end of cantilever beam by using wavelet coefficient singularity, which on the basis of spatial dimensionality reduction but not data reduction, and the value of wavelet coefficients becomes larger as it gets closer to the damage location.

## 4 Numerical simulation

**Fig 14:**
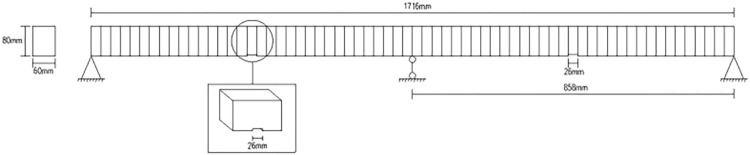
Damaged continuous beam model.

Through the experimental analysis of the fixed beam, it is verified that the wavelet coupling data method can effectively determine the damage position of the fixed beam structure. In order to further demonstrate the effectiveness of the data coupling method, the following numerical simulations of continuous beams will be performed and compared with the 1D wavelet method to evaluate the ability of the method to identify different damage locations and degrees. The continuous beam model is shown in Fig 14. Relevant parameters of continuous beam are as follows: span length of 1716mm, section size of *b*×*h* = 60*mm*×80*mm*, other parameters are shown in Table 1.

Wavelet toolbox is used for wavelet transform. In order to obtain the best wavelet coefficient graph, the suitable mother wavelet has to be selected. Research studies have shown that researchers choose the appropriate mother wavelet based on different criteria. Ovanesova and Suarez [34] selected the mother wavelet based on regularity, symmetry, and the ability to accurately reconstruct the analysis signal. Zhong and Oyadiji [35] selected the mother wavelet based on the number of vanishing moment and the effective support. However, in most cases, there are no fixed rules for the selection of mother wavelet, and the appropriate mother wavelet is based on different situations [36, 37]. In most cases, the trial and error method is applied to select a mother wavelet [38].

**Fig 15:**
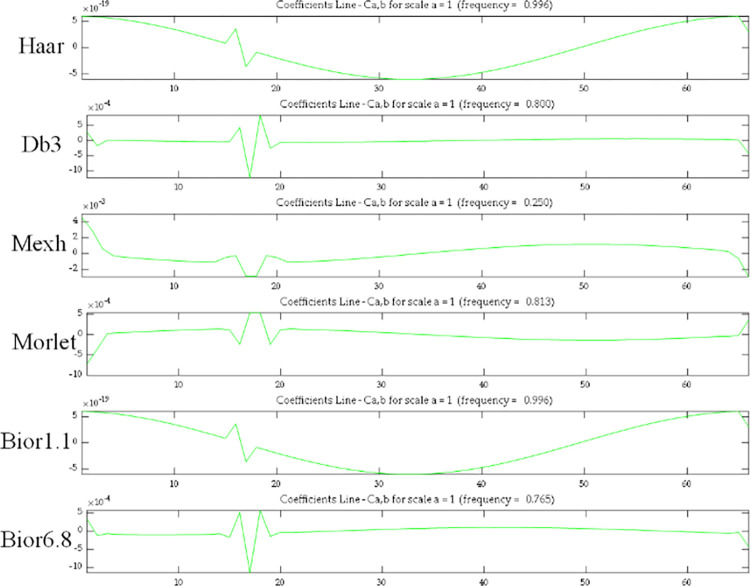
Coefficients of wavelet transform using different mother wavelets.

A continuous beam is numerically simulated to select the appropriate mother wavelet. The parameters of the beam are shown in Table 1. A 5% severity damage is set in element 17. The mother wavelet is selected as Haar wavelet, Db3 wavelet, Mexh wavelet, Morlet wavelet and Bior wavelet, and the strain mode of the beam is transformed by the above wavelet respectively. As can be seen from Fig 15, Bior6.8 can effectively identify damage and has no distortion at the end, so Bior6.8 is chosen as the mother wavelet.

### 4.1 Damage identification based on 1D-CWT of continuous beam

**Fig 16:**
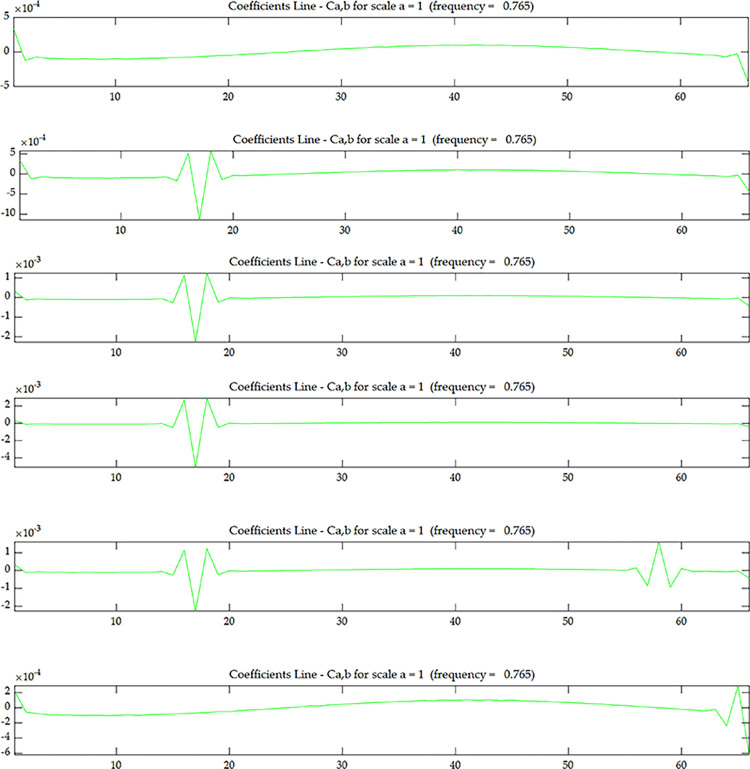
Wavelet coefficients of scenarios:(**a**) scenario 1;(**b**) scenario 2;(**c**) scenario 3;(**d**) scenario 4; (**e**) scenario 5;(**f**) scenario 6.

1D continuous wavelet transform (CWT) damage identification is to identify the damage by obtaining the position of the 1D strain mode singularities of the beam. The simple model of the beam is shown in the Fig 14. In Ansys19.2, BEAM188 is used to simulate the continuous beam element, and the damage was simulated by reducing the element stiffness. After the modal calculation of the model, the angular mode of the element node was obtained. The strain mode was obtained by the first order difference of the angular mode data, and the wavelet transform was carried out by BIOR 6.8 of wavelet toolbox, so as to obtain the wavelet coefficient of the strain mode. The damage severity of the continuous beam is simulated by stiffness reduction. Table 4 shows all the scenarios. Fig 16 shows the wavelet coefficients corresponding to each scenarios.

**Table 4:** Scenarios of continuous beam with transfixion damage.

Scenarios	Damage element	Damage severity (γ) (%)
1	none	none
2	17	5
3	17	10
4	17	20
5	17	10
	58	10
6	1	10
	65	10

In Fig 16(A), the wavelet coefficients of the non-destructive beam strain modes are relatively flat, and no obvious singularities are observed. The edge effect makes both ends protrusion, and the wavelet coefficients at both ends are basically equal. From Fig 16(B)–16(D), it can be seen that when the damage severity of element 17 is set to 5%, 10%, and 20%, respectively, their wavelet coefficient shows obvious singularities. In addition, with the increase of damage severity, the value of wavelet coefficient will also increase, because the increase of damage severity will increase the strain degree at the damage position, thus leading to the increase of wavelet coefficient.

Fig 16(E) shows the strain modal wavelet coefficients of the beam with both element 17 and 58 set to 10% damage severity. Compared with Fig 16(C), it can be concluded that the value of wavelet coefficients is not only related to the severity of damage, but also to the location of damage. Only relying on the same value of wavelet coefficients cannot prove the same severity of damage.

Fig 16(F) shows the strain mode wavelet coefficient of beam with damage at and near the end node. Although the wavelet coefficient value at node 1 fluctuates, it cannot distinguish whether it is caused by damage or edge effect. Node 65 is the element adjacent to the end point, and the obvious singularity can be seen from the figure, which proves that there is damage at point 65.

Through the above analysis, 1D-CWT can identify single damage and multiple damage along the length direction of continuous beam structure, but the specific spatial location of damage in the beam height direction cannot be determined, and the damage at the beam end cannot be effectively identified due to the existence of edge effect.

### 4.2 Damage identification based on W-DCM of continuous beam

**Fig 17:**
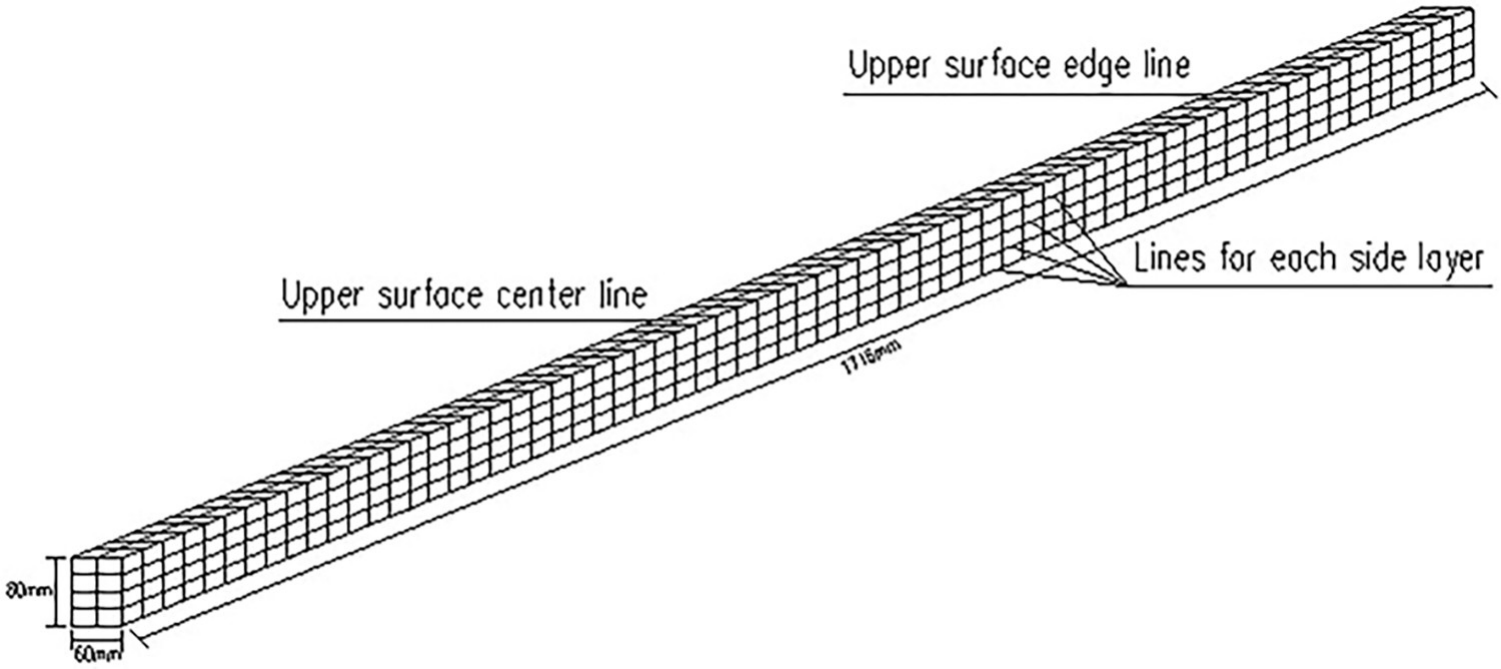
Schematic diagram of 3D mesh division of continuous beam.

The continuous beam is uniformly divided into 66 elements along the length direction, with the size of each element being 26 mm, and 4 layers along the height direction, each layer being 20 mm, and the schematic diagram of 3D element meshing is shown in Fig 17. By using Ansys finite element software, the displacement mode of the upper surface of the continuous beam is obtained, and then the corresponding strain mode is obtained by second-order differencing, and similarly, four layers of strain mode data along the length of the side of the beam are obtained, where *S*_1_ is the first layer of strain mode data near the upper surface, *S*_4_ is the fourth layer of strain mode data near the lower surface. The strain modal data *S*_*i*_ of other layers are numbered in ascending order. The *O*_*i*_ is obtained by normalizing and coupling the data according to Eqs (5) and (6), and then the continuous wavelet transform of the *O*_*i*_ is performed by bior 6.8 in wavelet toolbox, and the wavelet coefficient values can be obtained.

**Fig 18:**
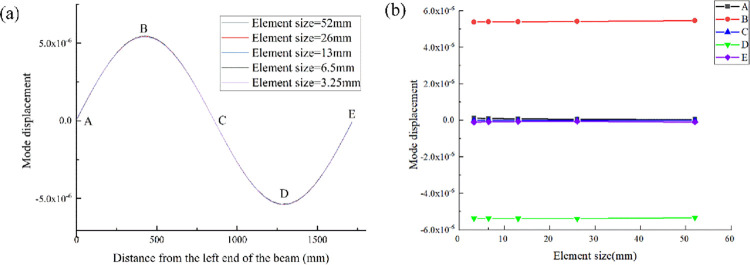
The first-order modes of continuous beams with different element size (**a**); Modal data at the same position of continuous beams with different element sizes(**b**).

Fig 18 shows the impact of element size on the first-order mode of the continuous beam model. It can be seen from Fig 18(A) that the first-order modes under these five element sizes are basically consistent, with a little dispersion at points B and D. Five points A to E are taken along the length direction of the beam, and the modal displacements of these five points are observed under different element sizes. It can be seen from Fig 18(B) that the modal displacement under the division of these five elements deviate very little and are basically in a straight line. Therefore, under the premise of ensuring efficiency and accuracy, the element size of 26mm is selected in this paper to divide beam elements.

The damage severity of the continuous beam is simulated by stiffness reduction. It can be seen from Eq (9) that the damage height can be used in numerical simulation to simulate the damage of the continuous beam. There are 4 scenarios as shown in Table 5. Scenario 4 contains two edge damage located in element 1 and 34 respectively.

**Table 5:** Scenarios of continuous beam with transfixion damage.

Scenarios	Damage element	Damage severity (γ) (%)	Damage depth *d* (mm)
1	none	none	none
2	17	4.9	1.35
3	17	9.7	2.76
	50	18.6	5.73
4	1	9.7	2.76
	34	9.7	2.76

**Fig 19:**
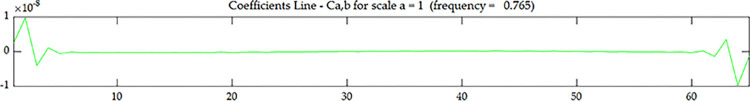
Wavelet coefficient of *U*^*C*^ under scenario 1.

**Fig 20:**
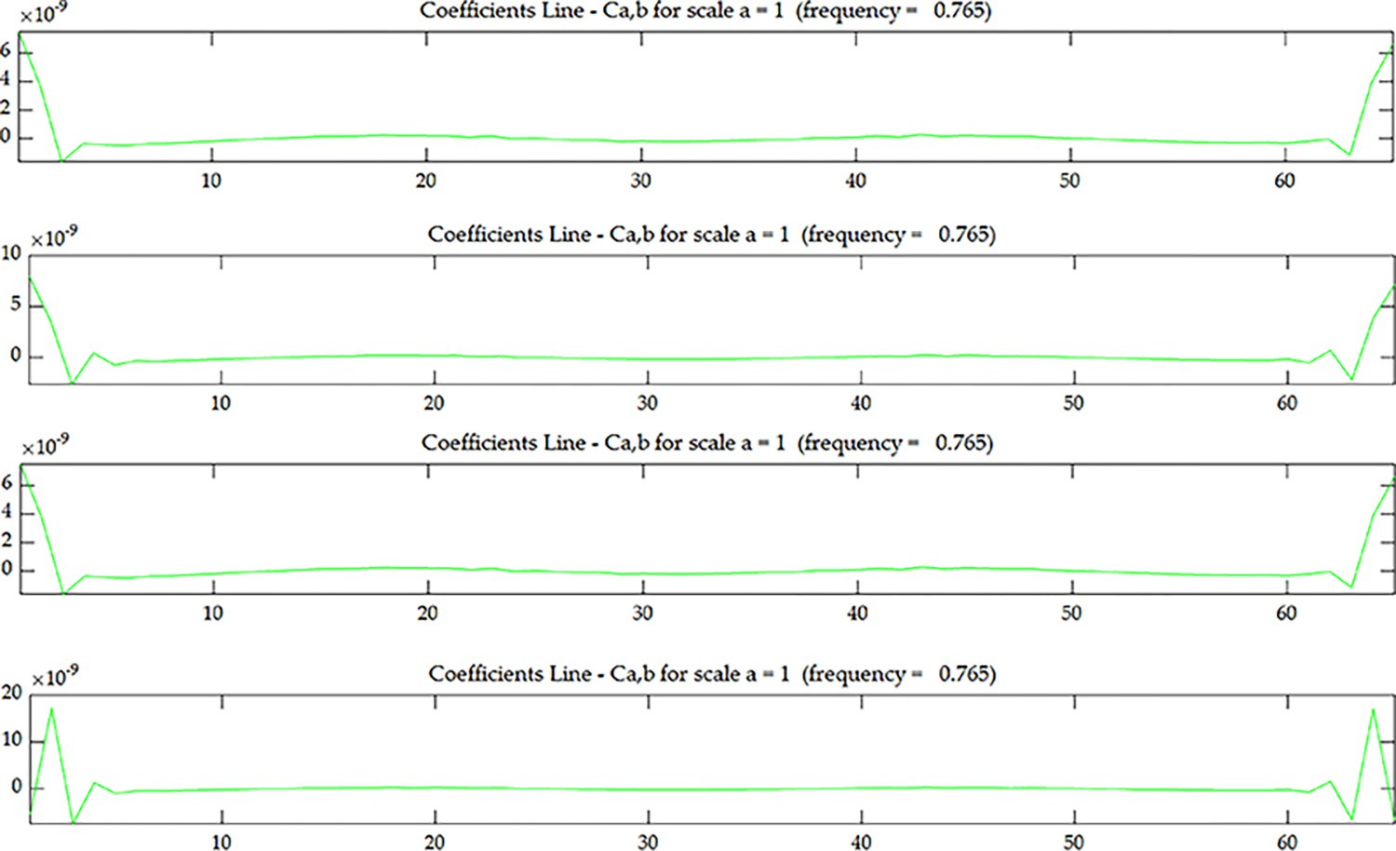
Wavelet coefficient of *O*_*1*_^*C*^~*O*_*4*_^*C*^ under scenario 1:(**a**) *O*_*1*_^*C*^; (**b**) *O*_*2*_^*C*^; (**c**) *O*_*3*_^*C*^; (**d**) *O*_*4*_^*C*^.

**Fig 21:**
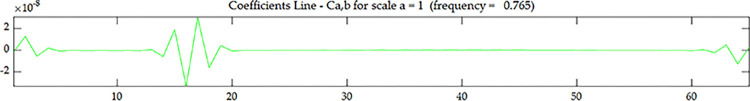
Wavelet coefficient of *U*^*C*^ under scenario 2.

**Fig 22:**
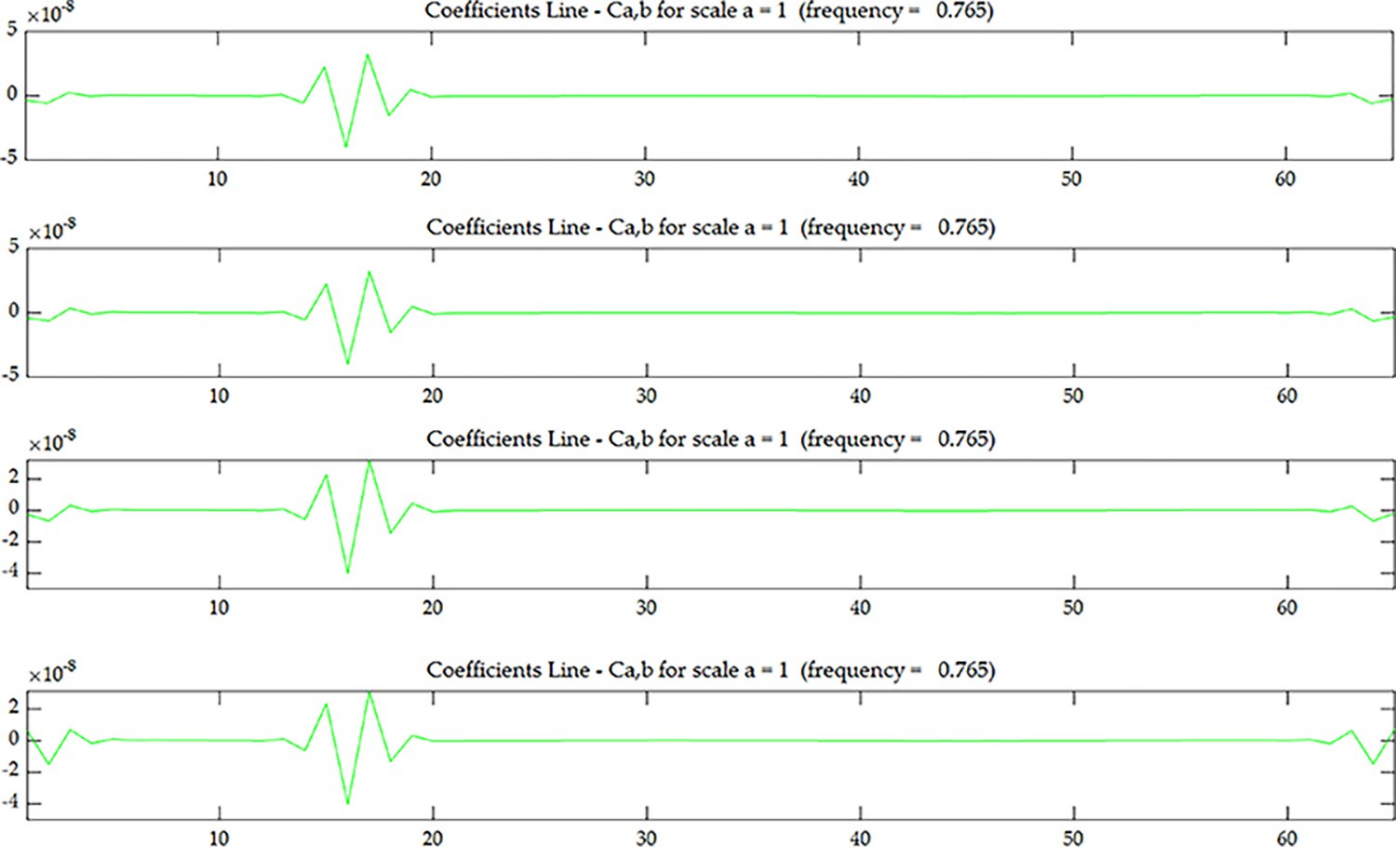
Wavelet coefficient of *O*_*1*_^*C*^~*O*_*4*_^*C*^ under scenario 2:(**a**) *O*_*1*_^*C*^; (**b**) *O*_*2*_^*C*^; (**c**) *O*_*3*_^*C*^; (**d**) *O*_*4*_^*C*^.

The coupled data *O*_*i*_ defined by Eqs (5) and (6) is used as the damage identification quantity to calculate its value. The wavelet coefficients of scenario 1 without damage and scenario 2 with single damage are shown in the Figs 19–22. It can be seen that the damage location is located at element 17 along the length of the beam, and the wavelet coefficient value at the damage location is increasing with the increase of the number of layers in the height direction. This indicates that the wavelet coefficient values are getting larger as the beam side gets closer to the damage location, and it can be seen that the damage occurs at element 17 along the length of the beam and in layer 4 along the height direction.

The wavelet coefficients at the nodes of the two ends of the continuous beam show large fluctuations due to the edge effect, but by comparing Figs 21 with 22, the edge effect of the wavelet coefficient map after coupling is smaller, which indicates that the coupled data method can also reduce the influence of the edge effect, and the later scenario 4 will explain in detail that the coupled data method can reduce the specific performance of the edge effect.

**Fig 23:**
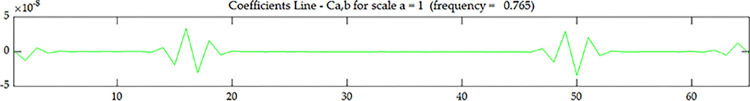
Wavelet coefficient of *U*^*C*^ under scenario 3.

**Fig 24:**
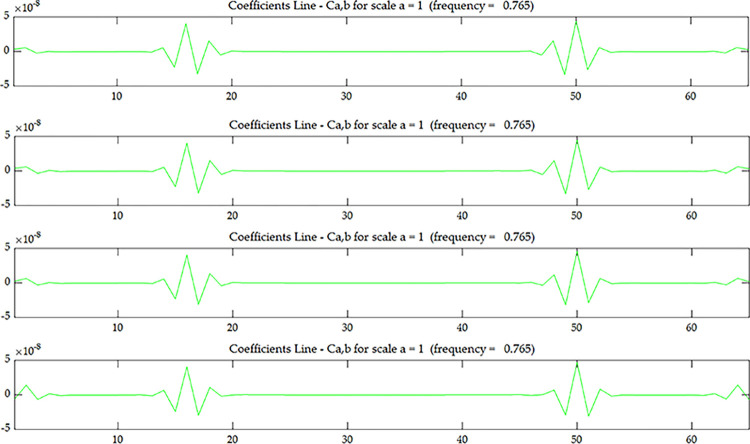
Wavelet coefficient of *O*_*1*_^*C*^~*O*_*4*_^*C*^ under scenario 3:(**a**) *O*_*1*_^*C*^; (**b**) *O*_*2*_^*C*^; (**c**) *O*_*3*_^*C*^; (**d**) *O*_*4*_^*C*^.

The coupling data *O*_*i*_ defined by Eqs (5) and (6) is used as the damage identification quantity to calculate its value, and the wavelet coefficient of the working scenario 3 containing two damages is shown in the Figs 23 and 24. It can be seen that the damages are located at element 17 and element 50 in the length direction of the beam, and the wavelet coefficient values of the damage location are getting larger as the number of layers increases. This indicates that the wavelet coefficient values are increasing as the side of the beam gets closer to the location of the damage, and it can be seen that the damage occurs at element 16 and element 51 along the length of the beam and at layer 4 along the height direction respectively, so that the spatial location of the damage is located. This shows that the coupled data method for two damage is able to effectively identify the damage location of a multi-damaged continuous beam.

**Fig 25:**
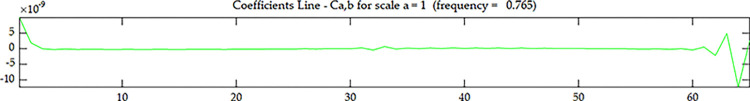
Wavelet coefficient using first-order mode shape of *U*^*C*^ under scenario 4.

**Fig 26:**
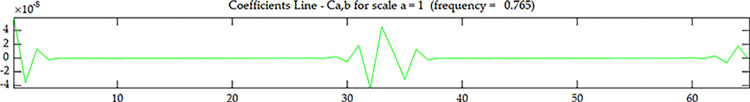
Wavelet coefficient using second-order mode shape of *U*^*C*^ under scenario 4.

**Fig 27:**
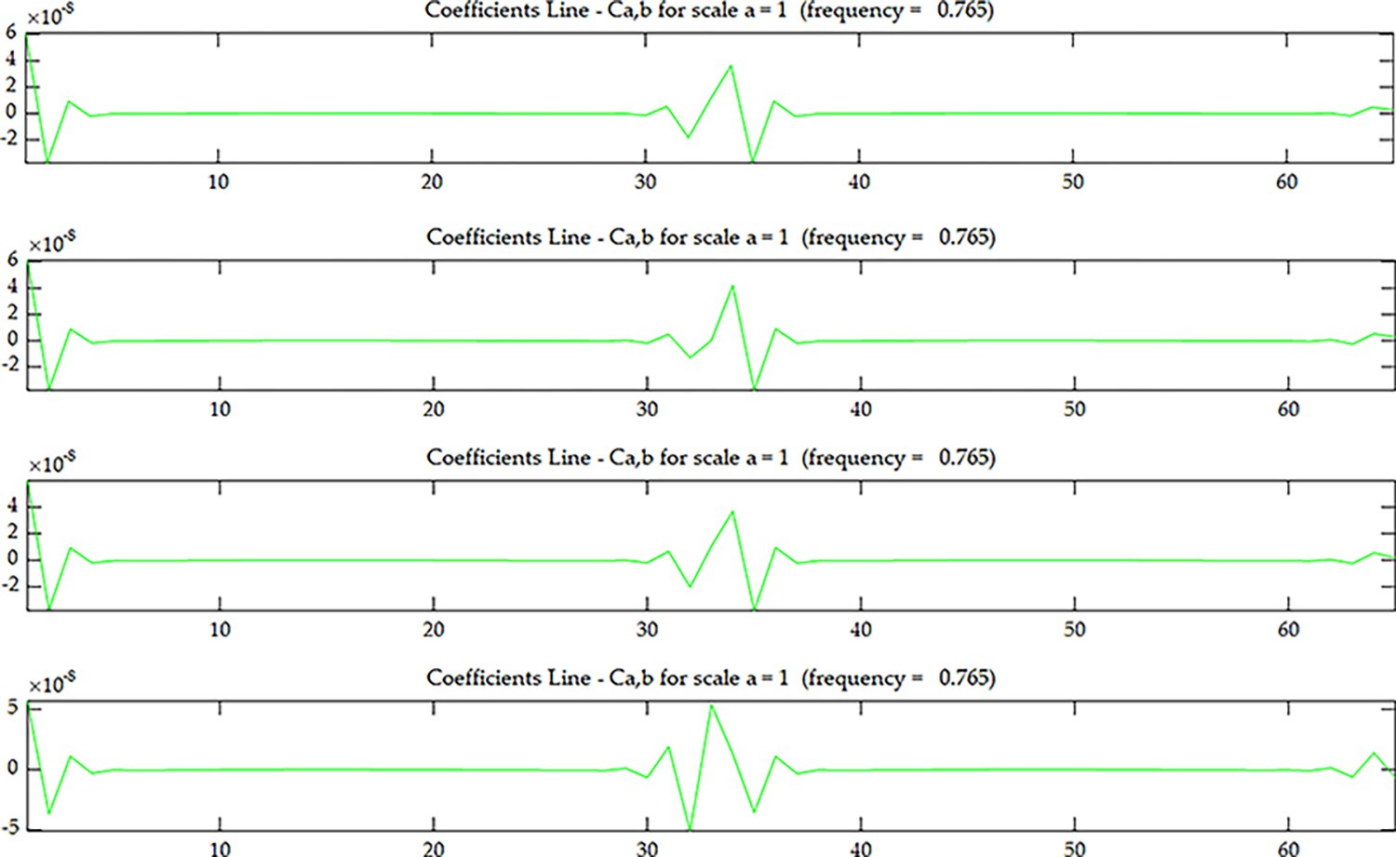
Wavelet coefficient using second-order mode shape of *O*_*1*_^*C*^~*O*_*4*_^*C*^ under scenario 4:(**a**) *O*_*1*_^*C*^; (**b**) *O*_*2*_^*C*^; (**c**) *O*_*3*_^*C*^; (**d**) *O*_*4*_^*C*^.

The coupling data *O*_*i*_ defined by Eqs (5) and (6) is used as the damage identification quantity to calculate its value, and the wavelet coefficient of the scenario 4 containing two edge damages is shown in Figs 25–27. According to the previous analysis, it can be found that if there is no damage at the end, the singularity caused by edge effect is symmetric or antisymmetric. As depicted in Fig 25, the utilization of first-order mode shape reveals that the singularities at both ends are inconsistent and the left end fails to satisfy the edge effect feature, indicating that damage is present near the left end of the support. However, due to limitations inherent in this mode’s characteristics, the damage near the middle support in scenario 4 is neglected.

As depicted in Figs 26 and 27, the situation changes when second-order mode is used. The wavelet coefficient of the right endpoint decreases significantly, while that of the left endpoint increases after coupling. This indicates that the left end of the beam is damaged while the right end is not. And the wavelet coefficient values of node 1 are getting larger as the number of lateral layers increases. Based on the preceding discussion, it can be inferred that there is damage present at the left end of the beam in element 1’s bottom section. Due to the second-order modes’ nature, a fluctuation symmetric about the middle support occurs here. However, as shown in Figs 26 and 27, the wavelet coefficient value at the middle support is asymmetric, indicating damage near this location. According to W-DCM, larger wavelet coefficient values indicate damage locations, and thus it can be concluded that element 34’s lower edge has sustained damage at its intermediate support.

## 5. Conclusions

In this paper, the spatial slicing method, which is often used in 3D signal processing, is applied to beam damage identification, and the wavelet based data coupling method(W-DCM) is proposed to identify structural damage. Through the experimental analysis of fixed beam and cantilever beam, and numerical simulation analysis of continuous beam, the results show that:

1. The 1D wavelet method can identify single damage and multiple damage along the beam length direction of continuous beam structure, but the specific spatial location of damage in the beam height direction cannot be determined, and the damage at the beam end cannot be effectively identified due to the existence of edge effect.
2. In the W-DCM, normalizing the data for the lateral layers amplifies the values at the damage location for each layer. Along the *h* direction, the closer to the damage location, the more pronounced the amplification effect of the values. The coupled values are also subject to this amplification effect. The coupled values determine both the damage location along the *l* direction and the *h* direction, thus obtaining the spatial location of the damage.
3. When the damage occurs at the fixed end of the beam, the W-DCM can increase the wavelet coefficient value of the damage point and reduce the wavelet singular value caused by the edge effect. The W-DCM can effectively avoid the edge effect and provide a new method for the identification of structural edge damage.
4. Through experiments and numerical simulations, the W-DCM can identify the spatial damage location of the fixed beam and continuous beam structure with damage severity of minimum 4.9%. However, the damage with severity of 9.7% near free end of the cantilever beam can not be recognized. Because of the characteristics of different modes, the second-order mode is more effective than the first-order mode in identifying the damage near the middle support of a continuous beam.

The need for a large number of highly sensitive sensors is one of the drawbacks of this approach. In future work, it is intended that the improved way to identify damage near the free end of beam, and to further validate the practical applicability of the proposed method.

## Supporting information

S1 Data (XLSX)
